# Stone Man: A Case Report

**DOI:** 10.5812/iranjradiol.10302

**Published:** 2012-12-27

**Authors:** Hamed Mortazavi, Majid Eshghpour, Mahdi Niknami, Morteza Saeedi

**Affiliations:** 1 Department of Oral Medicine, Faculty of Dentistry, Shahid Beheshti University of Medical Sciences, Tehran, Iran; 2 Department of Oral and Maxillofacial Surgery, Faculty of Dentistry, Mashhad University of Medical Sciences, Mashhad, Iran; 3 Department of Oral and Maxillofacial Radiology, Faculty of Dentistry, Tehran University of Medical Sciences, Tehran, Iran; 4 Department of Neurology, Faculty of Medicine, Mashhad University of Medical Sciences, Mashhad, Iran

**Keywords:** Myositis Ossificans, Ossification, Heterotopic, Complications

## Abstract

Fibrodysplasia ossificans progressiva (FOP) is a rare hereditary connective tissue disease characterized by the progressive ectopic ossification of ligaments, tendons, and facial and skeletal muscles throughout life. Symptoms begin in childhood as localized soft tissue swellings. Immobility and articular dysfunction appear with involvement of the spine and proximal extremities. The temporomandibular joint (TMJ) is a critical component involved in the maxillofacial region, resulting in severe limitation of masticatory function, although TMJ involvement is rare. The aim of this article is to present a 28-year-old man with dental problems and slowly progressive limitation of motion in the jaw, knees, shoulders and hips as well as neck distortion.

## 1. Introduction

Fibrodysplasia (myositis) ossificans progressiva (FOP) is a rare hereditary disease with progressive ossification of the muscles and connective tissue associated with pain and disability (1, 2). The incidence is approximately 1 in 2 million, with about 700 reported cases. No sexual, racial, or ethnic predilection has been reported (3). FOP usually begins in the first decade of life with an autosomal dominant trait and complete penetrance (4, 5). The FOP gen was discovered in 2006 by Shore et al. (6). This disease can lead to complete ossification of the muscular system and was first described in 1648 by Guy Patin as "stone man"(6, 7). Trauma to a region of the body may precede the development of a painful inflammatory mass which eventually calcifies. Even dental surgery may be a serious undertaking because it may trigger calcification of masseter or pterygoid muscles and consequently limit jaw mobility. Other abnormalities include microdactyly, hypoplasia and fusion of the digits, shortened metatarsal and metacarpal bones, microdactyly of the toe and thumb (8). There is no effective treatment for FOP (1, 9). Treatment of FOP may potentially be based on future interventions that block activin A receptor type 1 (ACVR1) also known as activin receptor-like kinase 2 (ALK2) signaling (9).

## 2. Case Presentation

**Figure 1:**
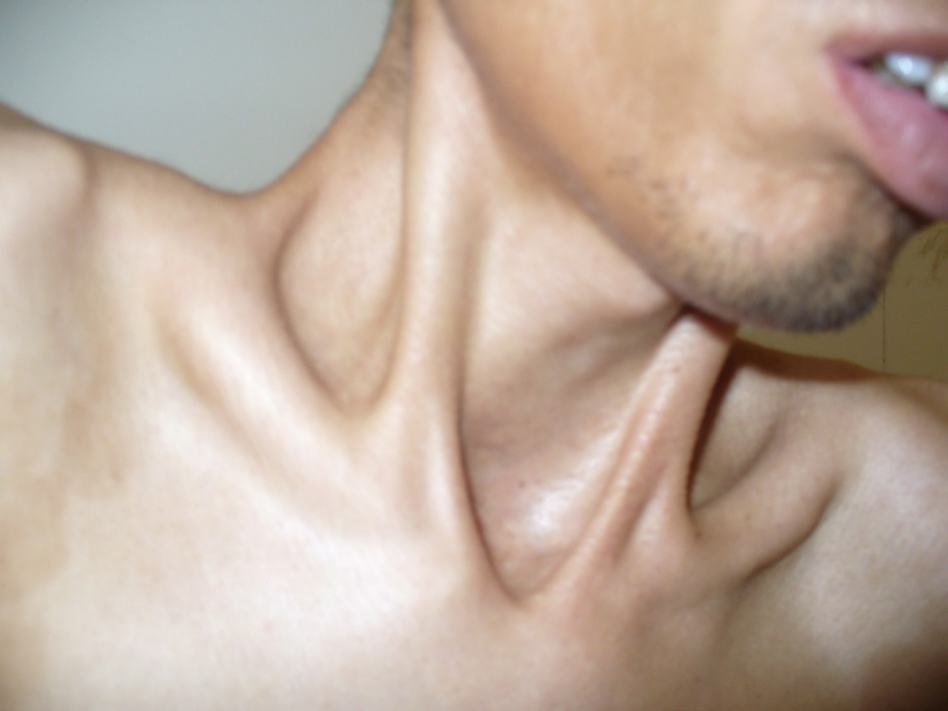
A 28-year-old man with progressive ossification of neck muscles.

**Figure 2:**
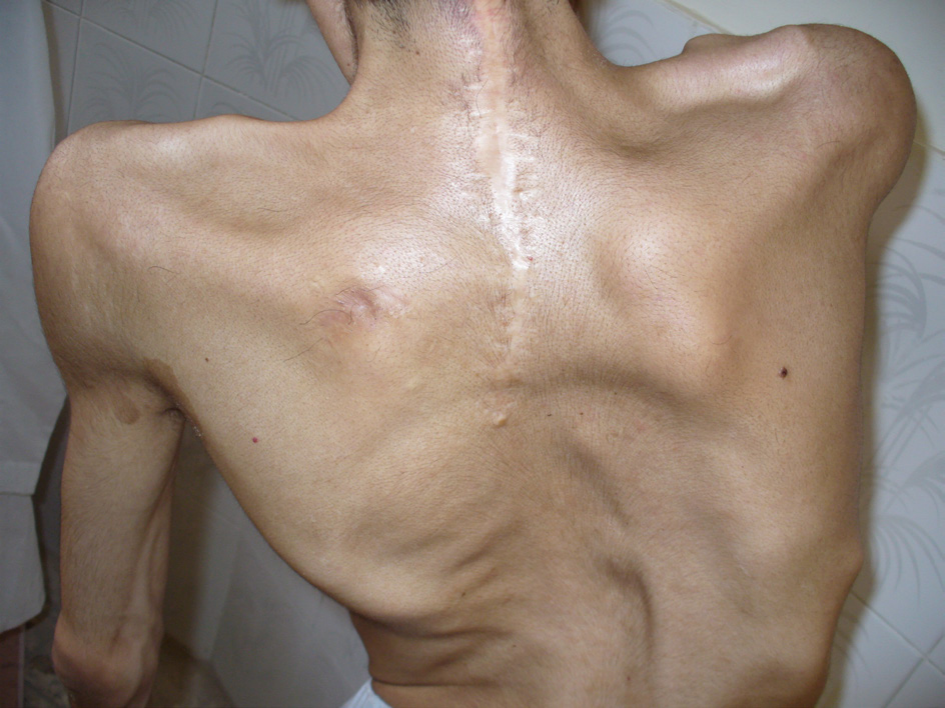
Body deformity and incision lines on the back of the patient after surgical procedures

**Figure 3:**
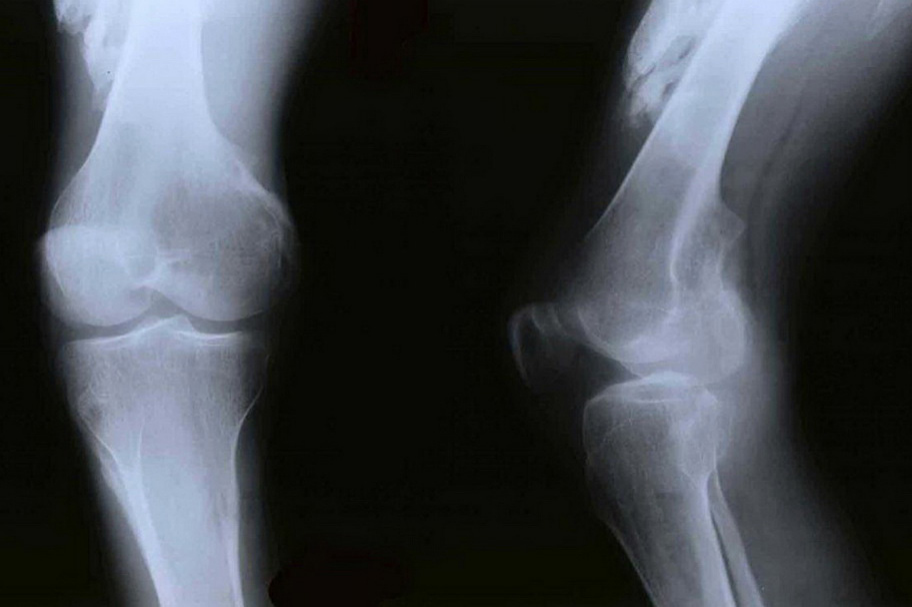
Lower limb radiographs showed ectopic ossification in the quadriceps muscle.

**Figure 4:**
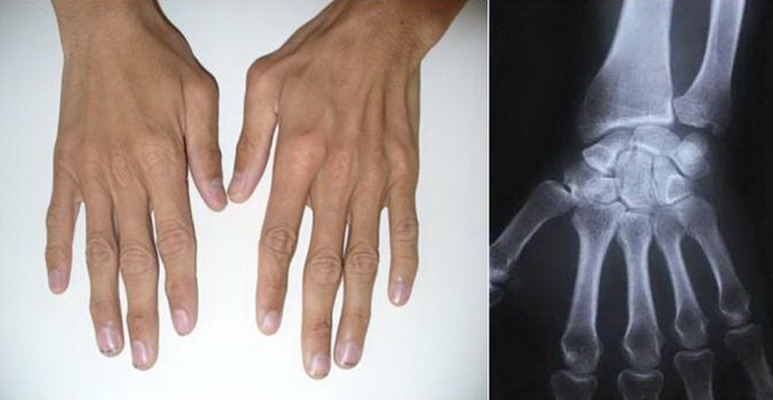
Hand radiograph and photograph showed shortening of the first phalynx of the thumb.

**Figure 5:**
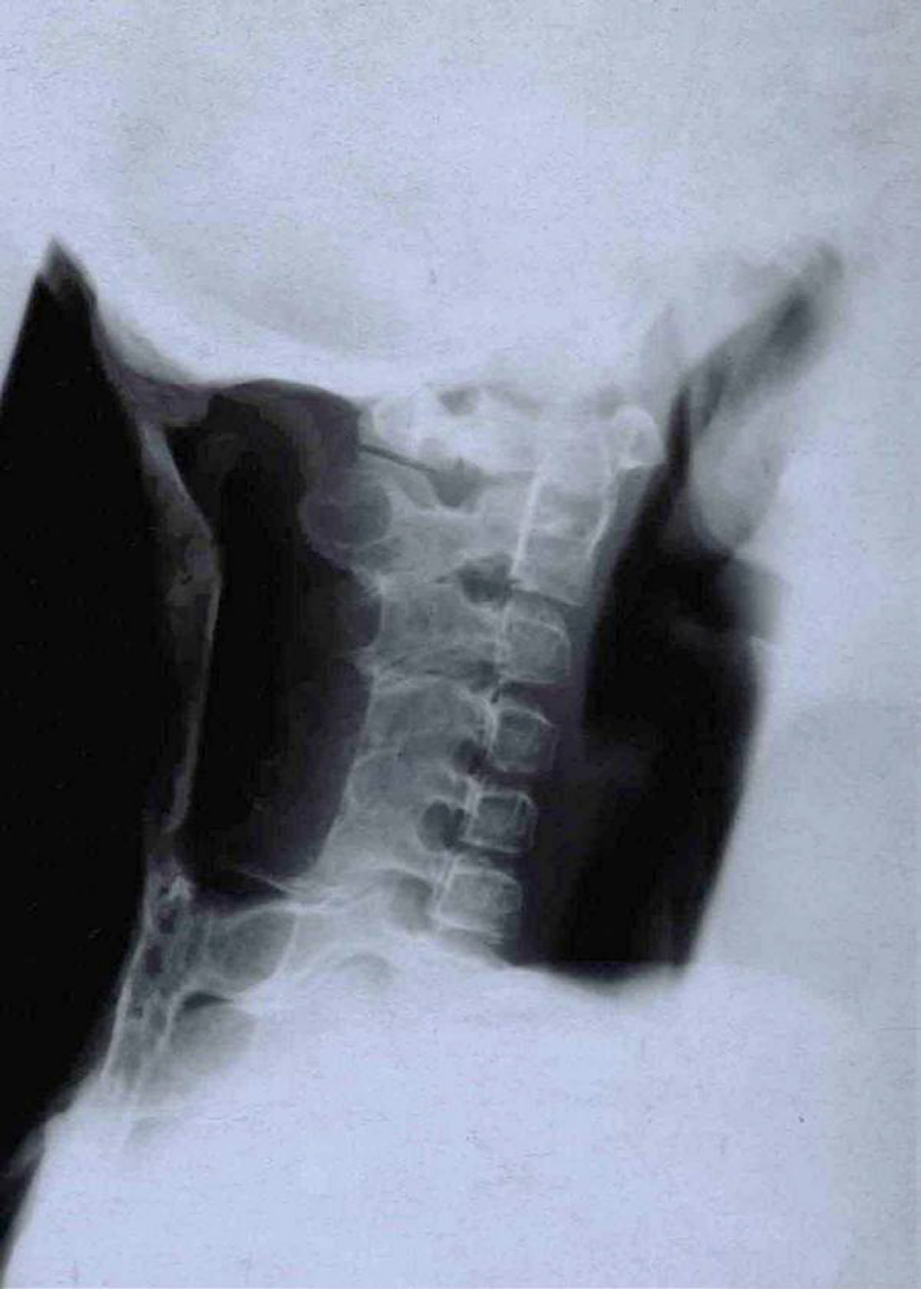
Lateral radiography of the neck showed ectopic ossification in the trapezius muscle.

A 28-year-old man (33.5 kg weight and 1.68 cm height) with progressive ossification of the muscles was referred to the department of maxillofacial surgery at the Dental Faculty, Mashhad, Iran because of dental abscess (Figure 1). He was diagnosed as a case of FOP. Based on medical history and documents, he presented the first symptoms of FOP as a painless mass on the scapula when he was three years old. After surgical removal of this mass, he experienced stiffness and slowly progressive limitation of neck motion. He had another surgery on the same region two months later because his complication returned. Over the next 20 years, his problems increased slowly as limitation of motion in the knees, jaws, shoulders and hips as well as neck distortion (Figure 2). At the age of 28, he had more functional disability. His last physical examination one week before the dental appointment showed a blood pressure of 130/60mmHg, a heart rate of 75/minute and respiratory rate of 20/minute. Musculoskeletal examination showed immobilization of the neck, jaw, spine, knees, shoulders and hips and incomplete extension of the elbow (Figure 3). Laboratory data (calcium, PT, PTT, urinalysis and blood cell count) were in the normal range. Radiography of the neck, spine, head, shoulder and feet demonstrated hereditary ossification. The patient’s hand radiograph and photograph showed shortening of the first phalynx of the thumb (Figure 4). The lateral neck radiograph also showed ossification of the trapezius muscle and complete fusion of the spine (Figure 5). Computed tomography (CT) of the paransal sinuses, mandible and maxilla showed no soft tissue mass lesion, focal bony lesion or abnormal calcification. Magnetic resonance imaging (MRI) of the neck showed no evidence of lymphadenopathy in the parapharyngeal space. MRI of the spine showed multiple ossifications of varying sizes along the paraspinal muscles. During dental evaluation, his maximum mouth opening was 5 mm and lateral jaw movement was about 0 mm. The patient had poor oral hygiene and multiple dental decays. Nine months ago he was visited by his dentist for severe infection in the right mandibular second molar. It was only treated by antibiotics, but after several days the infection involved the buccal space and leaded to buccal abscess. In our department, the abscess was drained and antibiotic therapy was carried out, but we could not extract the tooth.

## 3. Discussion

Guy Patin first described FOP in 1648 in a young man who “turned to wood”. The autosomal dominant inheritance of FOP was first described by Sympson in a case report of a seven-year-old boy with classic features of FOP (6, 7).

Despite growth in the knowledge of metabolic bone disease, little progress has been made in understanding the pathophysiology of FOP (1). Inaccurate diagnosis of FOP may lead to permanent injury and may change the natural history of the disease (10). The course of FOP is variable. Some patients develop FOP at an early age and die from starvation, infection or respiratory failure. Diagnosis of FOP is usually made based on the presence of two major criteria: malformation of the great toes and progressive heterotopic endochondral ossification. Laboratory tests may show a discrete increase of ESR during the “flare-ups” (11). However, most patients with FOP do not have an abnormal life span. Long periods of stable disease are common, but trauma is often a precipitating factor for disease reactivation. Surgery is hazardous in FOP because excision of the ectopic bone is commonly followed by accelerated ossification at the operation sites. In these patients, accurate oral hygiene is important and dental prophylaxis must be done from early dentition because injection of local anesthesia, especially mandibular block is an illicit method and all dental treatments should be done under general anesthesia (12). It should be considered that overstretching of the jaw for intubation during general anesthesia may cause additional trauma to the TMJ (temporomandibular joint) and may lead to disease flare-ups. General anesthesia in FOP patients should be accomplished by fiberoptic nasal intubation under light sedation. Superficial intravenous (IV) access and vein puncture is acceptable. Traumatic IV access and arterial punctures may cause heterotrophic ossification. This should be performed by well-trained anesthesia teams who are familiar with this type of procedures (13). The differential diagnoses of FOP include fibrosarcoma, extraosseous sarcoma, Still’s disease, ankylosing spondylitis, myositis ossificans circumscripta, and osteodystrophy (5, 12, 13). Other pathologies should also be considered in the differential diagnosis, including traumatic myositis ossificans and rigid spine syndrome (3, 8, 11, 14).

There is no known therapy effective against FOP. Medical therapy has limited success. Diphosphonates have been tried to reduce the ectopic calcification and to inhibit reossification following surgery. Retinoids, adrenocorticotropic hormone, corticosteroids, dietary calcium binders, intravenous infusion of ethylenediaminetetraacetic acid (EDTA) and warfarin are other measures that have been used without success (7).
